# The use of JAK inhibitors and tocilizumab in the management of TRAPS: a sibling case study

**DOI:** 10.1186/s12969-026-01225-4

**Published:** 2026-06-17

**Authors:** David Thai, Georgina Tiller, Jonathan Akikusa, Mark Taranto, Kathryn Shepherd, Seth L. Masters, Sam Mehr

**Affiliations:** 1 The Royal Children’s Hospital, Melbourne, Australia; 2 The Northern Hospital, Epping, Australia; 3 Monash Children’s Hospital, Clayton, Australia; 4 Centre for Innate Immunity and Infectious Diseases, Hudson Institute of Medical Research, Clayton, Australia

**Keywords:** TRAPS, Autoinflammatory, JAK inhibitor, Baricitinib, Tocilizumab, Needle phobia

## Abstract

**Background:**

Tumour necrosis factor (TNF) receptor-1 associated periodic syndrome (TRAPS) is an autoinflammatory condition. Most treatment options require regular injections, posing challenges for individuals with needle phobia.

**Findings:**

We reviewed the medical records of two siblings, a now 14-year-old female, and her 10-year-old brother, both diagnosed with TRAPS in early infancy. Both children responded to on-demand oral corticosteroid therapy, but due to frequent flares, persistent biochemical inflammation and poor growth, steroid-sparing therapies were required. Non-standard first-line therapies were subsequently selected as the eldest child had developed a needle phobia, and she was commenced on an oral Janus kinase (JAK) inhibitor. The younger brother was treated with tocilizumab, reflecting parental preference to minimise frequent injections. Over the last two years, both children have demonstrated significant improvement, with almost no further TRAPS flares, normalisation of baseline inflammatory markers and improvements in their growth trajectories. No adverse effects were reported on either agent.

**Conclusions:**

JAK inhibitor therapy and tocilizumab were effective in our two cases of TRAPS. To our knowledge, this is the first report describing the use of a JAK inhibitor in TRAPS, while also adding to the limited published experience with tocilizumab in TRAPS.

## Findings

## Background

Tumour necrosis factor (TNF) receptor-1 associated periodic syndrome (TRAPS) is an autosomal dominant, inherited autoinflammatory condition [1]. Uncontrolled systemic inflammation associated with TRAPS can result in secondary amyloidosis [2, 3]. Current standard treatments for TRAPS include corticosteroids, anakinra, canakinumab, and tocilizumab [2, 4–6]. Of these, corticosteroids are the only established oral option, with all other agents requiring injection. Janus kinase (JAK) inhibitors have not previously been reported in the treatment of TRAPS and may represent an additional oral therapeutic option [7].

## Methods

We reviewed the medical records of a now 14-year-old female, and her 10-year-old brother, each with a pathogenic variant in *TNFRSF1A*, [c.284 A > C (p.H95P)] and analysed previous treatments against the number of average disease flares, their inflammatory markers and growth, to gauge their effectiveness. Each child had responded to on-demand corticosteroid therapy, which was not considered a sustainable long-term solution. Due to an evolving needle phobia in the female child and parental reluctance for daily injectable treatments, we hypothesised that an oral JAK inhibitor or subcutaneous tocilizumab, could be therapeutic options that address the nuanced management challenges in these cases.

## Results

### Report A

A now 14-year-old girl, initially presented at 11 months of age with prolonged fevers and was initially treated as Kawasaki’s disease. Her early clinical course has previously been summarised [8].

At 17 months, genetic testing revealed a heterozygous variant in the *TNFRSF1A* gene (c.284A > C; p.H95P), confirming a diagnosis of TRAPS. Her father had similar fever episodes in childhood and had responded to on-demand prednisolone. Genetic testing confirmed both shared the same variant. She was commenced upon on-demand oral prednisolone (1-2 mg/kg/day; up to 5–7 days) for flare control.

At 3 years, due to increasing steroid dependency, etanercept (0.8 mg/kg/week) was commenced. Over the following 12 months, this became less effective with more frequent flares, complicated by inconsistent compliance due to an evolving needle phobia. This resulted in a parental preference to return to on-demand prednisolone therapy for flare management.

At 9 years, she commenced colchicine (0.25 mg [0.01 mg/kg/day], administered as half of a 0.5 mg tablet), but this was abandoned after three weeks due to gastrointestinal side effects.

At 10 years, she continued to have frequent fever flares, faltering growth and persistently elevated inflammatory markers, even outside of acute flares. Anakinra (3 mg/kg/dose) was commenced, but due to her needle phobia, consistent administration was not achievable, and treatment was discontinued after four weeks. At parental request, management reverted to on-demand prednisolone.

Her family sought alternative therapies, including hyperbaric oxygen therapy, and were resistant to non-steroid treatments for several years. She was referred to psychology services for her needle phobia but remained disengaged. At 13 years, given ongoing reluctance toward needle-based therapies, oral baricitinib was initiated (4 mg daily; ~0.1 mg/kg/day). Baricitinib is a non-selective JAK1/2 inhibitor [9] and was chosen over other JAK inhibitors due to its availability and clinical experience at our institution and published paediatric dosing guidance in STING-associated vasculopathy of infancy (SAVI) [10]. Although canakinumab is considered first-line therapy in TRAPS, it is not available at our centre due to its cost and was declined due to her needle phobia.

**Fig. 1:**
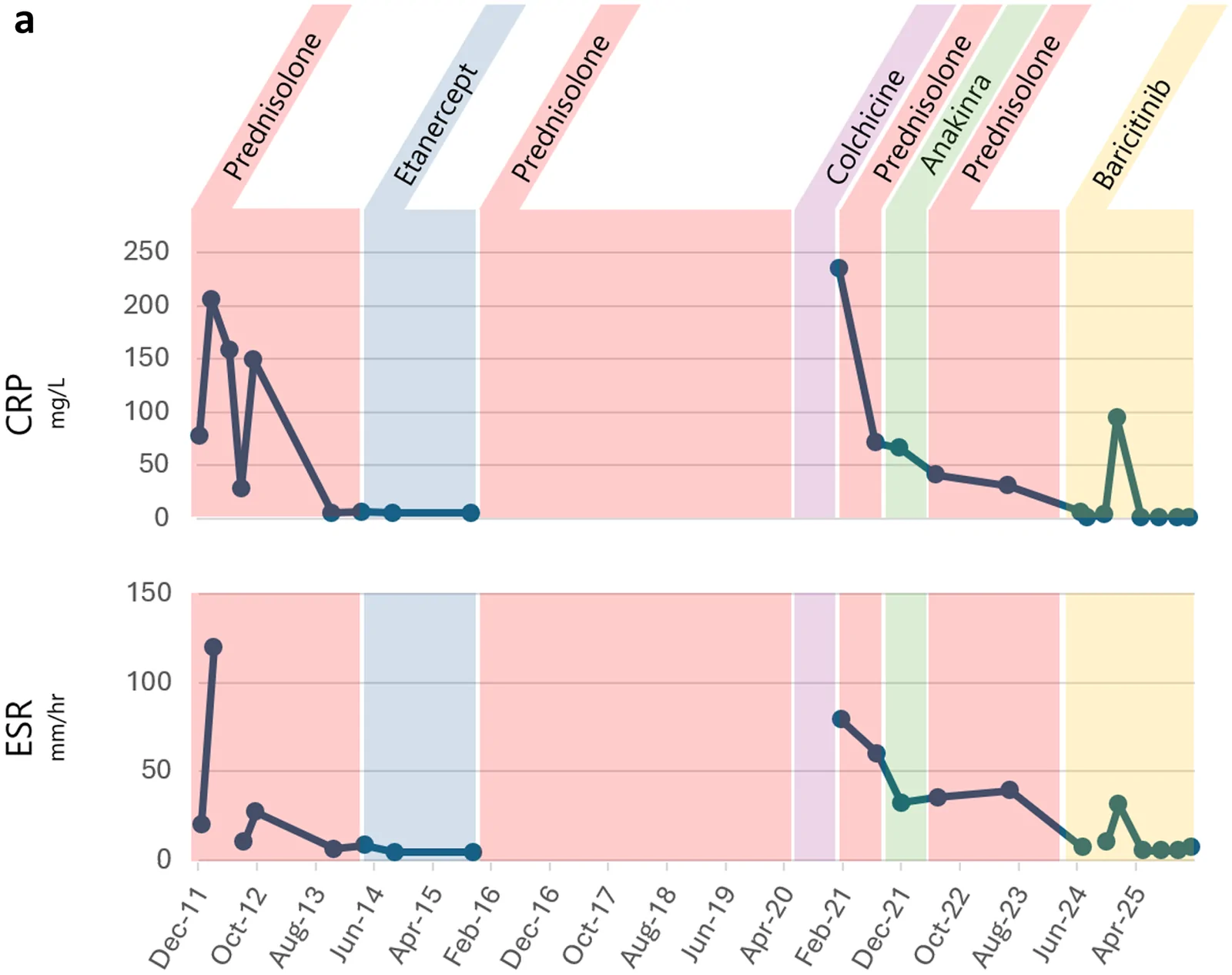

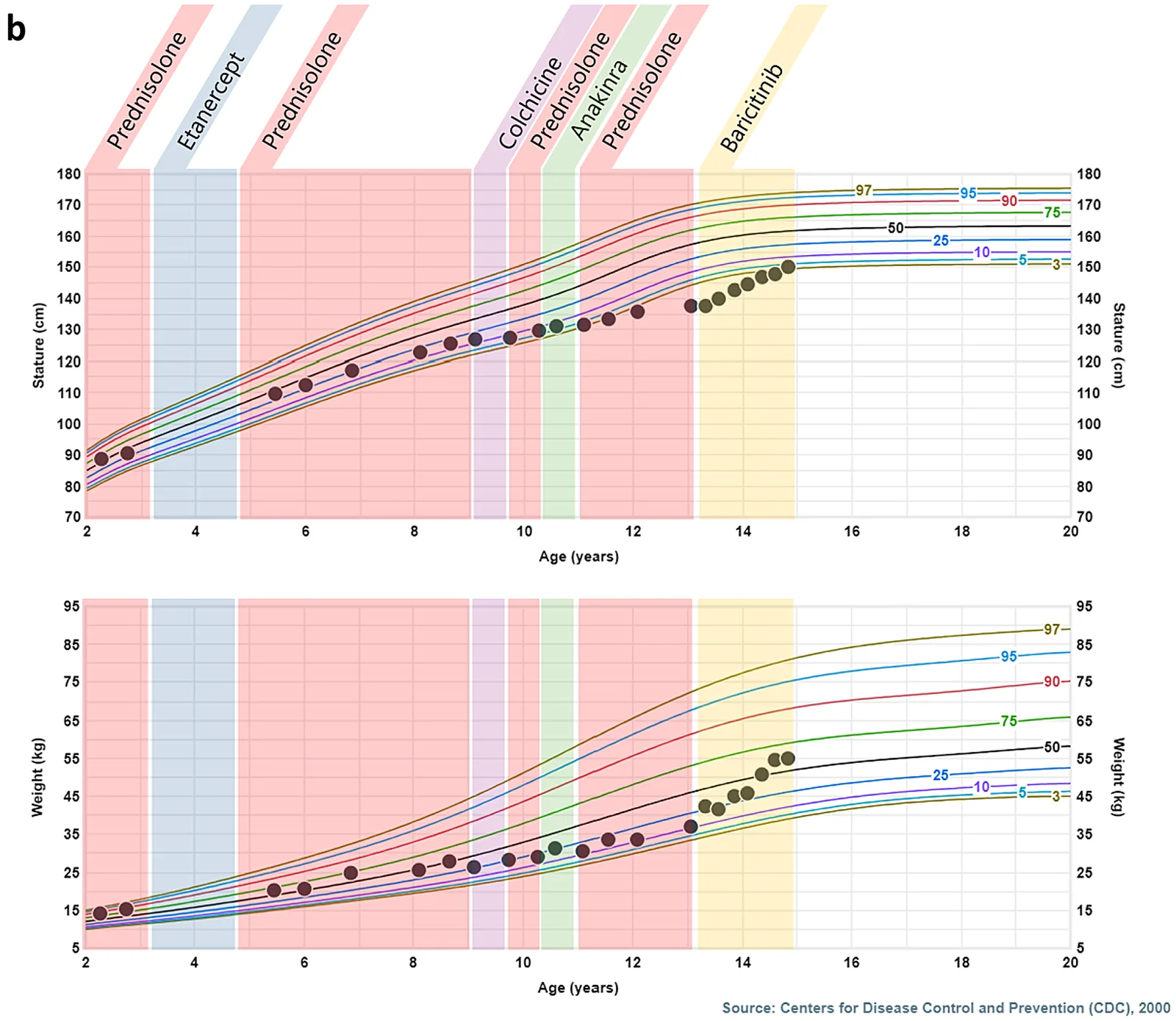
**a** Patient A – Inflammatory markers correlating with treatment. **b** Patient A – Growth charts correlating with treatment

Before baricitinib, she experienced 6–8 flares every six months since diagnosis. Following a daily dose of baricitinib 4 mg (0.1 mg/kg/day), the frequency of flares reduced to 2–3 episodes over the next six months. The dosage was increased to 4 mg twice daily (~ 0.2 mg/kg/day), leading to only one flare in the next six months, which did not require oral steroids. Following two years of baricitinib therapy, her inflammatory markers normalised (Fig. 1a), her growth trajectory improved (Fig. 1b), and no adverse effects (infections, cytopenias) have occurred.

## Report B

A now 10-year-old male, the younger sibling of Patient A, was diagnosed with TRAPS in infancy due to the known family history. He experienced fever episodes lasting 12 days, every 8 weeks, associated with faltering growth and raised inflammatory markers (CRP 181 mg/L).

He commenced on-demand prednisolone (1 mg/kg/day; up to 3–7 days for each flare), but due to ongoing flares, persistently elevated inflammatory markers and poor growth, he was commenced on colchicine at 6 years of age. This was abandoned within weeks due to diarrhoea.

**Fig. 2:**
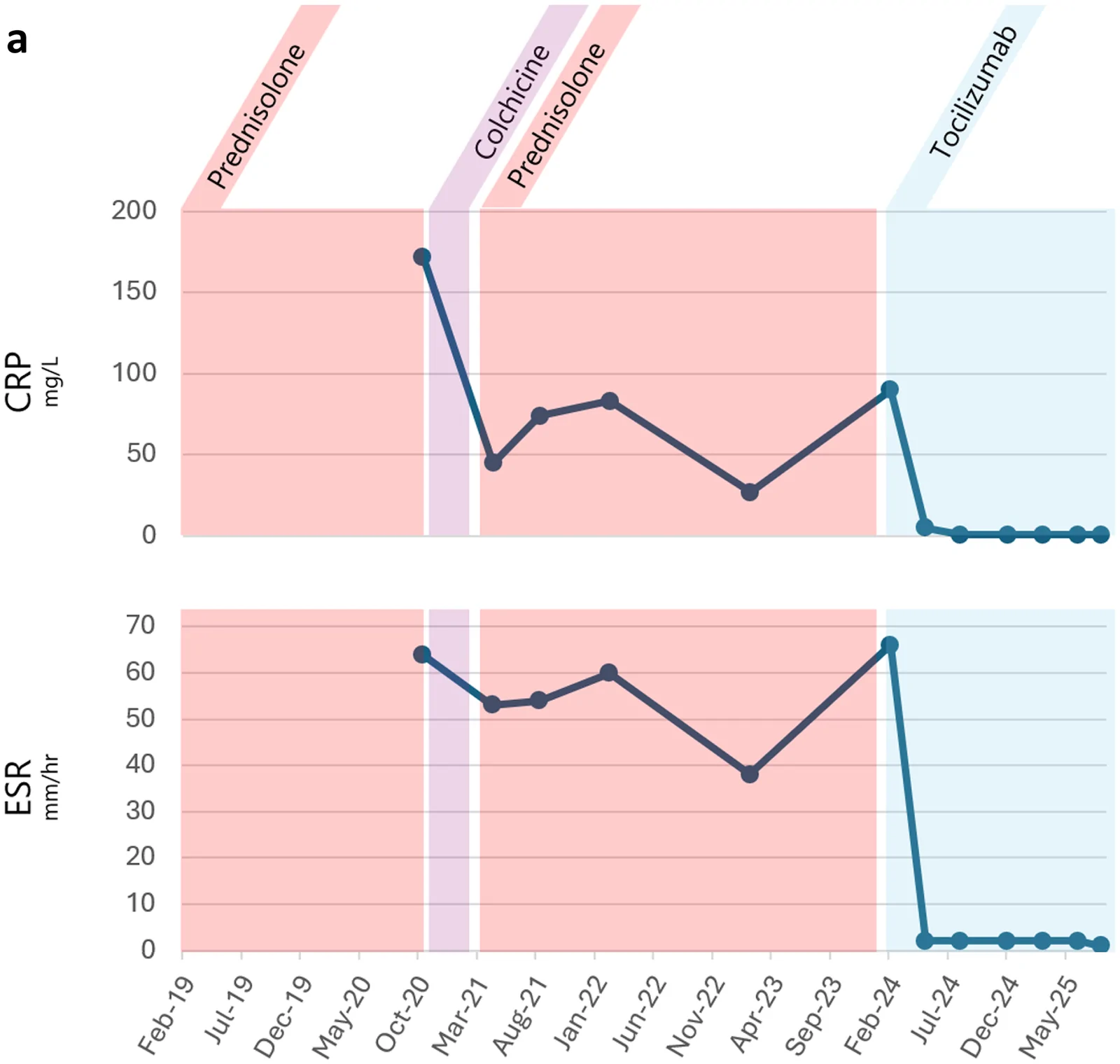

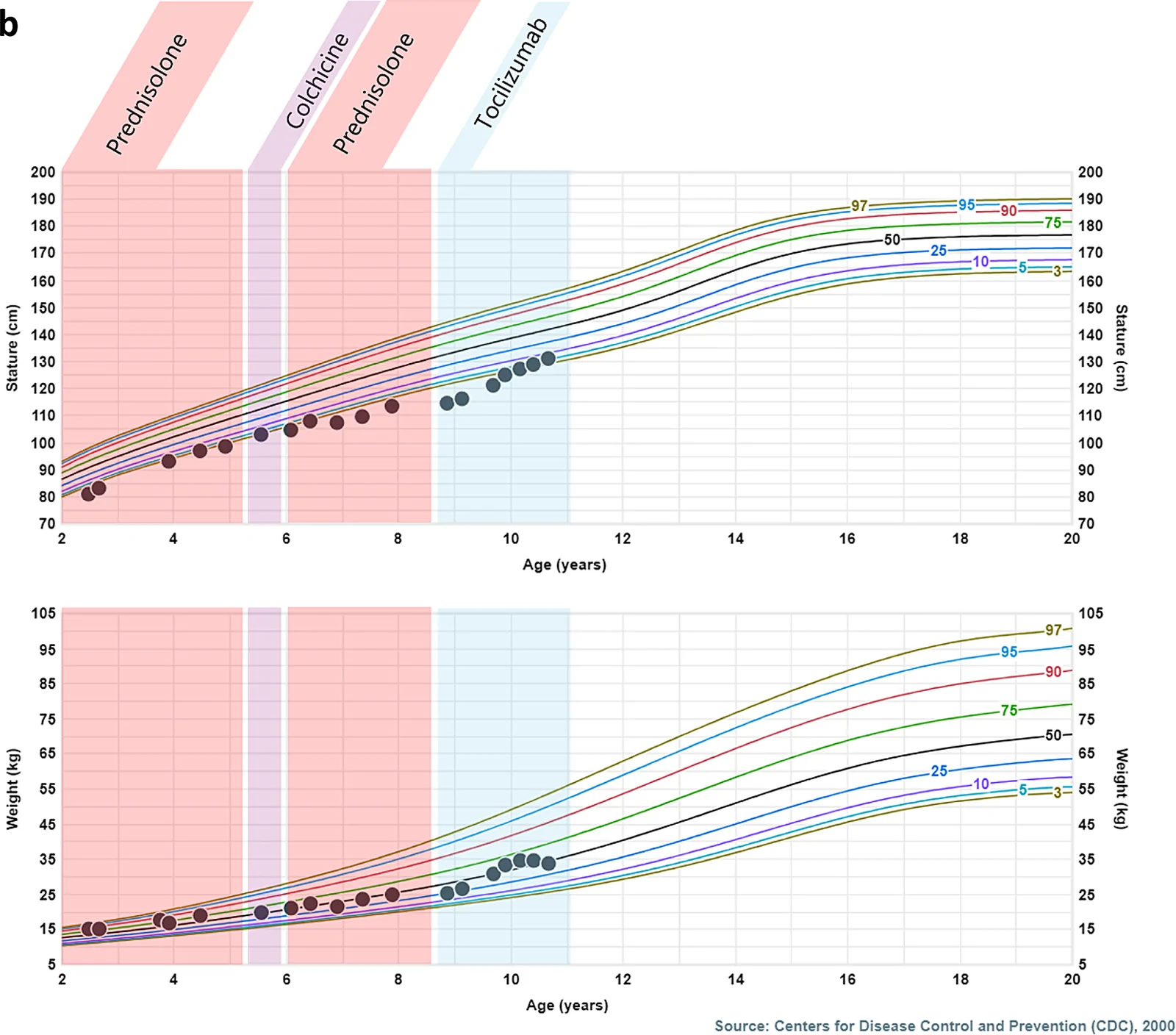
**a** Patient B – Inflammatory markers correlating with treatment. **b** Patient B – Growth charts correlating with treatment

At 9 years, he experienced persistent abdominal and joint pain with elevated inflammatory markers. Given his sister’s needle phobia, his parents were reluctant to use frequent injections. After being offered daily anakinra or fortnightly tocilizumab, his parents elected for subcutaneous tocilizumab. He was commenced on 162 mg (6.5 mg/kg, every two weeks). Before commencing tocilizumab, he experienced approximately three flares every six months, requiring on-demand oral steroids for almost all flares. Following two years of tocilizumab treatment, he has only had a single flare, with normalisation of inflammatory markers (Fig. 2a) and improved growth (Fig. 2b).

## Discussion and conclusions

The pathogenesis of TRAPS remains unclear; however, it is known that mutations within the *TNFRSF1A* gene are responsible for changes to the TNF receptor [1]. Early proposals described the inability to cleave the TNF receptor-1 upon stimulation with TNF-α, driving the autoinflammatory cycle [1]. Newer theories suggest that aberrant trafficking of the defective TNF receptor-1 results in inflammasome activation [11], or alternatively, that there is activation of the nuclear factor κB (NF-κB) and mitogen-activated protein kinase (MAPK) families [12], resulting in the overproduction of pro-inflammatory cytokines, including IL-1β, IL-6 and TNFα [12–14] which stimulate the secretion of serum amyloid-A (SAA) by the liver [13]. Excessive SAA deposition results in secondary amyloidosis, causing renal impairment [3].

Non-steroidal anti-inflammatory drugs (NSAIDs) and corticosteroids are accessible and often effective on-demand oral treatment options [2]. Long-term use is limited by their adverse effects. Oral colchicine is poorly efficacious, benefiting only 2/19 (11%) TRAPS patients [2].

Etanercept, a fusion protein comprising the extracellular domain of TNFR2 and the Fc portion of human IgG1, has been studied for its role in TNF-blockade in TRAPS patients [2, 15]. While etanercept has been shown to provide dose-dependent improvement in symptoms and inflammatory markers in TRAPS, responses are incomplete in some patients. This, along with injection site reactions are cited as the main reasons for discontinuation and subsequent switch to anti-IL-1β therapy [2, 15].

With the upregulation of IL-1β in TRAPS [13], several anti-IL-1 treatments have been used, including anakinra and canakinumab [2, 5]. Whilst now considered first-line therapies in TRAPS, these are limited to injectable forms and anakinra is associated with significant injection-site reactions [2]. Expert consensus recommends canakinumab as a first-line therapy [6, 14, 16], however it is not viable for needle-phobic individuals and, in some regions such as ours, is unavailable due to cost and lack of a compassionate access program.

Tocilizumab, a humanised monoclonal antibody targeting the interleukin-6 (IL-6) receptor, has shown variable efficacy in TRAPS [2, 4, 14, 17]. It is available only as an injection. In the first published case, tocilizumab (8 mg/kg every four weeks) was administered following failure of etanercept and anakinra [17]. After six months of treatment, no further TRAPS flares occurred, and corticosteroids were no longer required, despite persistent elevations in IL-1α, IL-6, and IL-8 levels [17]. Another case described identical dosing and demonstrated sustained clinical remission with normalisation of inflammatory markers, without symptom recurrence during therapy [4]. Although tocilizumab suppresses the hepatic production of acute phase reactants [4], thereby masking a CRP rise during disease flares, our patient also demonstrated other markers of efficacy, including reduced flare frequency, reduced need for on-demand oral corticosteroids and improved growth.

Cytokines relevant to systemic inflammatory diseases signal through pathways that converge on JAK-STAT activation [9]. Janus kinase (JAK) inhibitors are increasingly used in inflammatory conditions, particularly type-I interferonopathies, through inhibition of downstream signalling within the JAK-STAT pathway [7, 9], but are not otherwise used widely amongst monogenic autoinflammatory conditions. JAK inhibition has been reported to be effective in PAPA syndrome and colchicine-resistant familial Mediterranean fever (FMF), prototypic IL-18 and IL-1 mediated autoinflammatory diseases, respectively [7, 18, 19]. This suggests that autoinflammatory disorders may not be driven by a single cytokine pathway alone, but rather reflect broader inflammatory network activation.

Although direct reports of JAK inhibitor use in TRAPS are lacking, several aspects of TRAPS biology support a potential therapeutic role. IL-6 has been implicated in disease pathophysiology, and its signalling depends upon JAK-STAT activation [9]. Other cytokines involved in TRAPS, including IL-1β and TNF, may also be indirectly modulated by JAK inhibition. Emerging data suggest that baricitinib may reduce circulating levels of these cytokines, suggesting broader anti-inflammatory effects than previously appreciated [20].

Effective oral therapies remain important for patients unable to tolerate injections. Our two cases demonstrate that tocilizumab is a practical alternative to the daily injections required with anakinra, and that oral JAK inhibitors may be useful in TRAPS for patients with significant needle phobia. Both treatments were associated with fewer inflammatory episodes, normalisation of inflammatory markers, and improved growth trajectory, supporting their potential role as alternative therapies in TRAPS.

## Data Availability

No datasets were generated or analysed during the current study.

## Abbreviations

CRP: C-reactive protein
ESR: Erythrocyte sedimentation rate
IL: Interleukin
JAK: Janus kinase
MAPK: Mitogen-activated protein kinase
NF-κB: Nuclear factor κB
NSAID: Non-steroidal anti-inflammatory drug
SAA: Serum amyloid A
SAVI: STING associated vasculopathy of infancy
STAT: Signal transducer and activator of transcription
TNF: Tumour necrosis factor
TRAPS: TNF receptor associated periodic syndrome
